# Supplementary material for: Enhancing student career guidance and sentimental analysis: A performance-driven hybrid learning approach with feature ranking
Source: PLoS One. 2025 May 9;20(5):e0321108. doi: 10.1371/journal.pone.0321108 (PMC12064209; doi:10.1371/journal.pone.0321108)
Supplement: Attachment — Submitted filename: Reponse file.docx [file pone.0321108.s002.docx]

**Response File**

**Editor Comment**

**Comment 1.**

The images are blurry and unclear. Please provide higher quality versions of these figures (Figures 8-10)?

Answer: -	Response to Comment: The clarification regarding Figures 8-10, as referenced on the specified pages, has now been provided.

**Comment 2**

Benchmarking: Comparing your approach/model performance with only one study does not provide sufficient proof. I recommend benchmarking your model against several more recent studies for a more robust evaluation (Page 21).

**Answer:** - Benchmark models are provided as evidence, with a table illustrating dataset bias. Additionally, Figure 2 on page 22 is linked to this demonstration.

**Comment 3: -**

Table 10: My main concern is with the accuracy shown in Table 10. A 47% accuracy suggests a random result, which undermines the reliability of your findings. I suggest either redoing the experiment or excluding these results, as accuracy around 50% is typically considered random.

Conclusion: The conclusion needs significant improvement to better summarize the findings and implications of your study.

**Answer:** - Results with 47 percent accuracy have been presented, with graphs excluded throughout the study.

**Comments 4: -**

Proofreading: The paper requires thorough proofreading to correct any errors and improve overall clarity. Page 10, Page 17

**Answer: -**	Proof reading is done. Additionally, Figure 9, 10, 11 are removed for sake of reliability of the results, Table 5 and Table 10 both are removed from the paper.	Page 14, Page 18, Page 19

**Reviewer 1 comments and response of comments.**

**Comments 1	:**

Suggested Revisions, Include a detailed discussion on the limitations of the study and potential biases in the data Page number 20.

**Answer**	The study's limitations, focused on key features, skill set model, and range prediction, are discussed on page 21.

**Comment 2**

Can the model be validated with additional datasets for generalizability, and how does it compare with existing career prediction methods to highlight its improvements?

**Answer: -** On page 20, models or methods relevant to career prediction and their accuracies are presented, along with data to underscore the significant impact of the proposed method.

**Reviewer 2 comments and response of comments**

**Comment 1**

I think this is a good study. Long Captions for the figure should be added.

Answer: -	Long captions have been added for Figures 4, 7, 8, 11, 14, 16, and 19.

**Comment 2**

Figure 2 illustrates confusion but does not clearly convey how the data flows through the pipeline.

Answer: -	It would be beneficial to categorize the features into different groups. On page 5, features have been included in the diagram; however, there is limited space for additional details.
