# Supplementary material for: Enhancing student career guidance and sentimental analysis: A performance-driven hybrid learning approach with feature ranking
Source: PLoS One. 2025 May 9;20(5):e0321108. doi: 10.1371/journal.pone.0321108 (PMC12064209; doi:10.1371/journal.pone.0321108)
Supplement: Attachment — Submitted filename: Reponse File-Career Prediction Corrections.docx [file pone.0321108.s003.docx]

Rebuttal Letter

Dear Editor and Reviewer,

Find below the response to your potential comments and suggestions.

1. Could you please update your figure2's size ratio and Figure5 and Figure7's resolution as it is not very clear?

Response: Thanks for the suggestions, Figures 2, 5, and 7 are improved to be clearly visible.

2. For your average feature ranking, could you please explain more why you choose random forest tree here to calculate the feature important? Currently, there are many famous open source architecture like XGBM or lightGBM, which are also tree based structure and very good for feature importance selection. Why you didn't choose those known better performance algorithm in your architecture? What is the reason for choosing random forest tree here?

Response: Choosing Random Forest over XGBoost or LightGBM in our research article, we assume the following points:

- Simplicity and Ease of Use: Random Forest is generally easier to implement and tune than gradient boosting methods. It has fewer hyperparameters and is less sensitive to their values. This can be a valid justification if your research prioritizes simplicity and ease of use.
- Specific Dataset Characteristics: Certain dataset characteristics may favor Random Forest over gradient boosting methods. For example, Random Forest is known to handle high-dimensional data with many irrelevant features effectively. If the dataset exhibits such characteristics, this can support your choice.
- Computational Cost: Gradient boosting methods, particularly XGBoost and LightGBM, can be computationally expensive, especially with large datasets or complex hyperparameter tuning. If Random Forest provides adequate performance with significantly lower computational cost, this can be a strong justification, especially if computational resources are limited. (Anghel et al., 2018) (Daoud, 2019).
- Interpretability: Random Forest offers better interpretability than gradient boosting methods. We use feature importance scores from Random Forest to gain insights into the factors driving predictions. If interpretability is a primary goal of your research, this can justify the choice of Random Forest. (Couronné et al., 2018).

So, when we started our project 2 years ago, we considered the above features to select the Random Forest over other methods.

3. Based on suggestion 2 above, in your results evaluation section, could you please also compare the performance of your hybrid method with some famous architecture like XGBM or lightGBM? We wanna see if your hybrid method can outperform this architecture a lot in this specific issue.

Response: The comparison of XGBoost was conducted based on feature analysis, demonstrating that the proposed approach outperforms the state-of-the-art XGBoost technique in predicting students' career outcomes for clearer analysis. Figure 15 is added to show the comparison.
